# Social service organizations report improvements in social services-health care integration in survey during California’s medicaid initiative (“CalAIM”)

**DOI:** 10.1186/s12889-025-23419-3

**Published:** 2025-07-02

**Authors:** Abigail Arons, Priscilla Alban-Acuna, Melora Simon, John Whaley, Nicole Fossier, Amy Simon, Rohan Rastogi, Caroline Fichtenberg

**Affiliations:** 1 Division of General Internal Medicine at ZSFG, University of California San Francisco (UCSF), 2540 23rd St, San Francisco, CA 94110 USA; 2 Division of General Pediatrics, UCSF, 550 16th Street, Fourth Floor, San Francisco, CA 94158 USA; 3 Social Interventions Research and Evaluation Network, Department of Family and Community Medicine, UCSF, 675 18th Street, Floor 5, San Francisco, CA 94143 USA; 4 California Health Care Foundation, 1438 Webster Street, Suite 400, Oakland, CA 94612 USA; 5 Goodwin Simon Strategic Research, 4096 Piedmont Ave. #232, Oakland, CA 94611 USA

**Keywords:** Health related social needs, Social services organizations, Community based organization, Medicaid, CalAIM

## Abstract

**Background:**

To improve integration across health care and social needs, California’s Medicaid transformation initiative, CalAIM, depends on partnerships between health care entities and social services organizations (SSOs). Given the potential for power imbalance both within the landscape of SSOs and between SSOs and healthcare entities, monitoring and elevating SSO perspectives during implementation is paramount.

**Methods:**

We report the results of the SSO subgroup within a statewide survey conducted eighteen months into CalAIM implementation (July-September 2023, following CalAIM’s January 2022 launch) among a diverse convenience sample of SSO respondents who were familiar with CalAIM. Results were analyzed using multivariable logistic regression models with CalAIM participation and six measures of perceived improvement as outcomes.

**Results:**

There were 355 SSO employee respondents representing 51 of 58 California counties, 66% of which worked in SSOs contracted as CalAIM providers. CalAIM participation was negatively associated with operating in a single county (OR 0.47, 95%CI 0.24–0.95), and positively associated with pilot participation (OR 6.4, 95% CI 2.5–16.4) and prior managed care contracting experience (OR 2.5, 95% CI 1.4–4.7); pilot participation was negatively associated with fewer employees and non-profit status, indicating a possible mediation effect. Across all six models with outcomes of perceived improvement in health care-social services integration, CalAIM participation was strongly associated with increased odds of perceived improvement (OR 2.28 to 4.97), as was being frontline staff or a manager compared to senior leadership (Manager OR 1.76 to 2.85; frontline OR 1.92–3.35), and being a housing services provider (OR 1.80 to 2.15).

**Conclusions:**

Our analysis suggests that among SSOs familiar with CalAIM, smaller SSOs were less likely to be contracted CalAIM providers, possibly mediated by lower rates of prior Medicaid contracting experiences (e.g. pilot participation). Contracted SSOs perceived improvements in health care-social services integration at both the patient and organizational levels, suggesting CalAIM is functioning as intended in the perception of SSOs. We demonstrate a feasible method for ongoing monitoring of the SSO perspective that can inform near-term policy directions to ensure small SSOs with close community ties are fully included as partners in CalAIM.

**Supplementary Information:**

The online version contains supplementary material available at 10.1186/s12889-025-23419-3.

## Introduction

With increased attention to the role of health related social needs (HRSN) in driving health outcomes, state initiatives to integrate health care and social services have grown exponentially over the past decade [1]. The California Advancing and Innovating Medi-Cal (CalAIM) program is a comprehensive Medicaid transformation initiative with a goal “to create a more coordinated, person-centered, and equitable health system” [2]. It includes, among other elements, high-touch case management across health care and social services organizations (SSOs) (“Enhanced Case Management”), and direct contracting between managed care plans and SSOs for housing supports, food, and other specified social services (“Community Supports”) for certain groups of Medicaid enrollees. Details on eligibility, coverage, and payment for CalAIM services have been described elsewhere [2]. CalAIM is currently operational, having launched in January 2022, building upon two previous pilot initiatives that began in 2017, Whole Person Care and Health Homes [2]. While aspects of CalAIM’s financing authorities are unique, partnerships between Medicaid managed care plans and SSOs to address HRSN exist in at least 30 states [3].

CalAIM and similar efforts hinge on partnerships between SSOs and health care entities, predicated on the idea that SSOs are both closely tied to the communities they serve and bring expertise in social service provision [4, 5]. In California and other states, concerns have arisen about the reality of health care-SSO partnerships given the power imbalance within traditional health care payment structures between health care and SSOs, and the wide variation among SSOs ranging from small, local non-profits to multi-state corporations. Gathering and elevating SSO perspectives throughout implementation of HRSN partnership initiatives is key to monitoring whether these concerns are playing out and provides an avenue for SSO input.

Prior studies have garnered SSO perspectives in the planning and implementation stages of health care-SSO integration initiatives. These include qualitative studies with interviews with health care and SSO leaders in California (prior to CalAIM implementation) [4, 6] and other states [7–13], a brief, small sample survey [6, 13] and analysis of public comments on Medicaid waivers [12]. These studies reveal broad support among SSOs for integration initiatives [4, 6, 8, 12–14] and a variety of implementation lessons and challenges [4, 6, 7, 9, 10, 12–14]. An additional study reviewed CalAIM SSO contracts for signs of unequal participation [5]. While some studies focused mainly on SSO’s technologic and logistic capabilities, in those that assess the potential for health care-SSO power imbalance specific concerns arising include that health care and plan perspectives may overshadow SSOs [7, 8] that larger SSOs may predominate among contracted SSOs, rather than smaller organizations with close community ties [4, 5], and that both forces may particularly exclude SSOs serving marginalized communities that experience the brunt of structural racism [4–6]. These studies have been limited to smaller sample sizes (*n* < 100). Surveys can permit sampling a larger array of perspectives. No study has used a longer survey to assess SSO perspectives, nor assessed SSO perspectives during CalAIM implementation.

*Purpose*: We report the SSO results of a mid-length, statewide survey eighteen months into CalAIM implementation, using CROSS Survey reporting guidelines [15]. Our main objective was to answer two questions from the perspective of SSO employees: (1) What are the characteristics of SSOs who are aware of CalAIM but choose not to participate? (Hypothesis: smaller SSOs and/or SSOs that focus on serving Black, Indigenous and other people of color (BIPOC) may choose to participate CalAIM at lower rates than other SSOs) (2) From the SSO perspective, has there been improvement in integration and coordination across Social Services and Health Care since CalAIM, and what are the SSO characteristics associated with positive perceptions?

## The current study

### Background

We conducted an online, cross-sectional survey of a statewide convenience sample of SSO employees familiar with CalAIM, in July-Sept 2023, eighteen months after CalAIM’s launch. The SSO module was fielded within a larger survey of CalAIM stakeholders that also included health care providers, managed care plan employees and others [16]. The survey development and fielding effort was led by the California Health Care Foundation and Goodwin Simons Strategic Research, with assistance from UCSF and San Diego 211. The SSO module was funded by a grant from the Robert Wood Johnson Foundation. This work was considered not to be human subjects research and certified as Exempt under the University of California San Francisco IRB #23-38573. There were no competing interests among the research team or funders.

### Recruitment and sampling

#### Study population

Survey participants were individuals reporting current employment at an SSO operating in California. Individuals were excluded if they reported “no familiarity” with CalAIM. In the initial survey process, participants were also excluded if they reported serving fewer than 30% Medicaid patients; this exclusion criterion was subsequently lifted for the majority of the survey period. Multiple respondents from a single organization were possible.

#### Sample size

To ensure a margin of error of +/- 5 points at the 95% confidence level, we determined that a sample size of at least 350 responses would suffice [17]. 

#### Recruitment

The survey link was widely distributed through channels identified by the research team and key informants as broadly reaching SSOs. These included sector-specific email listservs, newsletters, webinars and other events hosted by CalAIM learning collaboratives and technical assistance providers, state agencies, social services associations, and community foundations. Regional and service-type representation of survey respondents was monitored; in cases of underrepresented regions and service types, the research team conducted internet searches (e.g. “county name” & “service type”) and emails with survey links were sent to organizational email addresses and leadership email addresses for identified organizations. In addition, a company with a proprietary list of SSOs promoted the survey. Participants received $25 for their time.

#### Sample characteristics

The target population was people working at SSOs that provide services related to HRSN in California. Given the lack of a centralized list of SSOs in California, characteristics and size of the target population were unknown.

#### Survey administration

All respondents completed the survey via an online link (Forsta Survey software, 2023) active from July to September 2023. The survey software uses cookies as well as HTML5, ETag, and Flash unique identifiers to help prevent duplicate responses. Responses that appeared not to be valid (e.g., very short completion times, nonsensical open-ended responses) were excluded.

#### Measure

The SSO module consisted of up to 62 questions with skip logic (Appendix Table 1). Topics were generated from themes in 6 focus groups with CalAIM implementers (conducted Winter/Spring 2023) and approximately 10 semi-structured key informant interviews conducted by the research team with technical assistance providers, state officials, and academic evaluators. Survey items were drafted by the research team and revised with key informant input. The SSO module was pilot tested with 4 SSO employees who completed a near-final version of the full survey and provided written feedback, which the research team incorporated in the final version.

**Table 1:** Characteristics of SSO survey respondents by CalAIM participation status (*n* = 355)

Characteristic	% overall sample	Not CalAIM Contracted (*n* = 120)	CalAIM Contracted* (*n* = 235)	*p*-value
Indicators of Smaller Organization and/or Close Community-Ties				
*Under 50 FTEs (vs. >=50)*	42%	48% (54)	38% (84)	0.085
*Operate in one county only (vs. >1)*	73%	83% (100)	68% (159)	0.002
*Nonprofit (vs. private/govt.)*	82%	87% (104)	79% (186)	0.083
*Any BIPOC/NEL specialty (vs. none)*	33%	35% (42)	32% (75)	0.559
Previous Organizational Experience				
Pilot participant**	31%	7% (8)	44% (103)	< 0.001
*Prior MCP contracts*	51%	36% (37)	69% (145)	< 0.001
*Prior HIE/CIE participation*	66%	49% (59)	75% (176)	< 0.001
Services Provided				
*Housing/Homelessness*	70%	55% (66)	77% (182)	< 0.001
*Food-related services/Food assistance*	52%	51% (61)	52% (123)	0.788
*Information & Referral services*	52%	53% (63)	51% (121)	0.857
*General social services assistance*	50%	46% (55)	52% (122)	0.278
*Benefits navigation*	43%	38% (46)	45% (105)	0.253
*Services for older adults or people with disabilities to live in the community*	36%	28% (34)	40% (93)	0.037
*Recuperative care/Medical respite*	21%	3% (3)	31% (72)	< 0.001
*Re-entry services following incarceration*	21%	15% (18)	23% (55)	0.064
*Child welfare services*	19%	15% (18)	21% (49)	0.183
*Home modification services*	14%	9% (11)	17% (39)	0.057
*Sobering center/Sobering services*	12%	4% (5)	17% (39)	0.001
*Legal services*	10%	10% (12)	9% (22)	0.847
*Asthma remediation services*	8%	1% (1)	11% (27)	< 0.001

*CalAIM contracted includes contracting with a MediCal Managed Care Plan as an Enhanced Case Management (ECM) provider, Community Supports provider or both. Of the 235 contracted SSO respondents, 45% [*n* = 106] were both ECM and Community Supports providers, 45% were [*n* = 106] only Community Supports providers, and 10% [*n* = 23] were only ECM providers

**Pilot participation indicates the SSO participated in either Whole Person Care or Health Homes pilot programs or both

### Analytic approach

#### Independent/Explanatory characteristics

We examined three sets of SSO characteristics: (1) indicators of organization size and population specialization (fewer than 50 full time employees [FTEs], whether the organization operates in only one county, nonprofit status, and whether the organization reported any specialization in BIPOC and/or Non-English Language [NEL] populations), (2) prior organizational experiences (pilot participation, prior MCP contracting, and prior health information exchange [HIE] and/or community information exchange [CIE] experience) and (3) types of services provided as they pertain to Community Supports and/or ECM populations of focus (e.g., housing, food, re-entry services, etc.).

#### Dependent variable/outcomes

CalAIM participation was defined as being contracted as a provider of Enhanced Care Management and/or Community Supports. To evaluate SSO perceptions of improvement in health care-social services integration under CalAIM, we used six measures of integration (Patient access to and coordination of comprehensive services including for social needs, SSO’s ability to manage patients’ needs and coordinate with other organizations, SSO’s technologic infrastructure and financial stability; see Appendix Table 1 for full text), measured on a Likert scale, which we dichotomized to “better” or “much better” versus “no change,” “worse,” or “much worse”.

#### Data analysis

We conducted bivariate analyses between all SSO characteristics and CalAIM participation using chi-squared tests. We then developed a multivariable logistic regression model using all variables significantly associated (*p* < 0.05) with CalAIM participation in the bivariate analysis, and the four predictors pertaining to organization size and population specialization regardless of bivariate results. Within each domain we evaluated multicollinearity and conducted sensitivity analysis using interaction terms and determined all variables to be independent except providing housing services and recuperative care services (we kept housing services as this was more frequently reported). To assess possible mediation by pilot participation, we conducted a second multivariate model with Pilot Participation as the dependent variable, limited to single-county SSOs in counties that were pilot counties, using the same set of independent variables as the main model (except prior MCP experience and prior HIE experience because we could not determine if these pre-dated the pilots).

For the perceptions outcomes, we followed a similar approach, first carrying out bivariate analyses of associations between each of the 6 outcomes and the same explanatory variables (except prior MCP contracting experience as this would not affect perceptions of service coordination). We added CalAIM participation and respondent role type (which could affect perceptions) as explanatory variables in this model. We then constructed six multivariate logistic regression models, using the same set of independent variables in all 6 multivariate models for consistency (any variables that met *p* < 0.1 with at least 4 outcomes in the bivariate analysis, and all four indicators of organizational size and population specialization).

Given the possibility that there were multiple respondents from the same SSO which would cause non-random error, we conducted a sensitivity analysis using mixed effects logistic regression at the county level (organization name was not collected, and county was the nearest hierarchical level available) with the final logistic regression models for both the participation and perceptions outcomes. All analyses were conducted using Stata (V18; College Station, TX).

#### Missing data, non-response error

Explanatory variables had no missingness except: FTEs (*n* = 24 missing), prior MCP contracting (*n* = 44), respondent role (*n* = 13), and county (*n* = 8). For outcomes, CalAIM participation had no missingness; the perceptions questions had missingness rates ranging from 14% (*n* = 48) to 21% (*n* = 76). Respondents missing perceptions outcomes were not more likely to be smaller or BIPOC-specialized.

## Results

The recruitment yielded a convenience sample of 531 SSO respondents. Of these, 18% (*n* = 97) were excluded (*n* = 24 excluded for not serving 30% or more Medicaid patients prior to that criterion being lifted, *n* = 73 excluded for “no familiarity” with CalAIM) and 15% (*n* = 79) dropped out with most questions incomplete. This resulted in a final analytic sample of 355 SSO respondents. Given that the survey was widely disseminated through many channels, we do not know how many people received the survey link and were unable to calculate a response rate.

### Respondent characteristics

Respondents worked in 51 of 58 California counties, and 66% worked in SSOs holding contracts with managed care plans to deliver ECM and/or Community Supports (“CalAIM Contracted”; *n* = 235). In terms of demographics, respondents were mean age 45 (SD 12.3, Range 21–82), 63% Female, and identified as: White (52%) Hispanic/Latino/a/x (27%), Black (10%), Asian (5%), American Indian/Alaska Native (2%), Native Hawaiian/Pacific Islander (1%), with 13% declining to answer race/ethnicity. 20% (*n* = 70) of respondents were frontline staff, 41% (*n* = 145) managers, and 36% (*n* = 127) senior leadership. The most frequent services provided were housing (70%, *n* = 248), food (52%, *n* = 184) and information and referral services (52%, *n* = 184), with 78% (*n* = 278) reporting more than one service type.

### Research question #1: what are characteristics of SSOs associated with CalAIM participation?

Compared to SSOs who were familiar with CalAIM but chose not to participate, in bivariate analyses, CalAIM-contracted organizations were less likely to operate in only one county (68% vs. 83%, *p* = 0.002), to have fewer than 50 FTEs (38% vs. 48%, *p* = 0.085), and to be a nonprofit (79% vs. 87%, *p* = 0.083). CalAIM participating SSOs were also more likely to have been a pilot participant (44% vs. 7%, *p* < 0.001), to have had prior contracts with plans (69% vs. 36%, *p* < 0.001), to have previously participated in an HIE or CIE (75% vs. 49%, *p* < 0.001), and to be a provider of the services paid for as part of Community Supports (housing/homelessness services (77% vs. 55%, *p* < 0.001), services for older adults or people with disabilities (40% vs. 28%, *p* = 0.037), recuperative care/medical respite (31% vs. 3%, *p* < 0.001), re-entry services (23% vs. 15%, *p* = 0.064), home-modification services (17% vs. 9%, *p* = 0.057), sobering services (17% vs. 4%, 0.001), and asthma remediation services (1% vs. 11%, < 0.0.001)) (Table 1).

CalAIM participation was not associated with whether an SSO had any BIPOC or NEL specialization, nor in a sensitivity analysis defining this variable as SSOs reporting only one BIPOC specialty. In the sensitivity analysis with mixed effects clustered on county to account for multiple respondents from the same SSO, the *p* values maintained significance with the exception of housing provider.

In the multivariable analysis (Table 2), CalAIM participation was negatively associated with operating in a single county (OR 0.47, 95%CI 0.24–0.95). Whether an organization had fewer than 50 FTEs, was a non-profit, or reported any BIPOC/NEL specialty were not significantly associated with CalAIM participation. Both pilot participation (OR 6.4, 95% CI 2.5–16.4) and prior MCP contracting experience (OR 2.5, 95% CI 1.4–4.7) statistically significantly increased odds of CalAIM participation and prior HIE/CIE experience also trended toward a positive association (*p* = 0.07). Of service types, only providing housing services statistically significantly increased odds of CalAIM participation (OR 1.92, 95% CI 1.04–3.60).

**Table 2:** Multivariate logistic regression analysis of factors associated with odds of CalAIM participation among the full SSO sample (*n* = 355)

Organizational Characteristic	OR (95% CI)
Under 50 FTEs (vs. >=50)	1.27 (0.70–2.31)
Operate in one county only (vs. >1)	**0.47 (0.24–0.95)**
Nonprofit org (vs. private/govt.)	1.27 (0.53–3.04)
Any BIPOC/NEL specialty (vs. none)	0.95 (0.52–1.74)
Pilot participant (vs. not)	**6.91 (2.70-17.68)**
Prior contracts with MCPs (vs. none)	**2.60 (1.40–4.83)**
Prior HIE/CIE participation (vs. none)	1.79 (0.94–3.40)
Housing services	1.92 (1.04–3.60)*
Services of community living	0.85 (0.44–1.63)
Sobering services	2.19 (0.72–6.61)
Asthma remediation services	5.77 (0.65–51.14)

Bold indicates statistically significant at *p* < 0.05. Prior HIE/CIE had *p* = 0.07

*In the sensitivity analysis with mixed effects at the county level, housing services changed from *p* = 0.04 to *p* = 0.08

In the model with pilot participation as the outcome, for single-county SSOs operating in pilot counties (*n* = 209), the odds of pilot participation were statistically significantly lower for organizations with under 50 FTEs (OR 0.42, 95% CI 0.19–0.94) and nonprofits (OR 0.15, 95% 0.04–0.54), indicating a possible mediation effect (Appendix Table 1).

### Research question #2: what are SSO perceptions of SSO-health care coordination under CalAIM and SSO characteristics associated with positive perceptions?

In bivariate analyses, operating in a single county and being a non-profit were statistically significantly associated with perceptions of improvement in at least 4 domains, as were pilot participation, CalAIM participation, prior HIE/CIE participation, and being a provider of housing, recuperative care, sobering, or asthma remediation services (Appendix Table 2). In the multivariate analysis (summarized in Table 3, full results in Appendix Table 3), having fewer than 50 FTEs was associated with higher odds of perceived improvement in patient access to services (OR 1.72, 95% CI 1.01–2.92), and patient coordination of services (OR 1.82, 95% CI 1.07–3.08). Non-profit status was associated with lower odds of perceived improvement in patient access to services (OR 0.43, 95% CI 0.20–0.91) and IT infrastructure (OR 0.44, 95% CI 0.21–0.91). Being in a single county and specializing in BIPOC/NEL populations were not statistically significantly associated with perceived improvement in any domain.

**Table 3:** Summary of six multivariate logistic regression models with evaluating association between independent variables and odds of perceived improvement in six areas (“much better” or “Better”) since CalAIM

	Patient *access* to services (inc. social needs)	Patient *coordination* of services	SSO’s ability to manage patients’ needs	SSO’s coordination with other organizations	SSO’s IT infrastructure	SSO’s financial stability
Under 50 FTEs (vs. >=50)	**1.72**	**1.82**	1.49	1.21	1.24	1.31
Operate in one county only (vs. >1)	0.76	0.78	0.87	0.75	0.82	0.96
Nonprofit org (vs. private/govt.)	0.43*	0.62	0.55	0.51	0.44*	0.60
Any BIPOC/NEL specialty (vs. none)	0.76	1.14	1.35	1.27	0.87	1.26
CalAIM Participant (vs. not)	**3.61**	**3.69**	**4.19**	**2.28**	**4.72**	**4.97**
Prior HIE/CIE participation (vs. none)	1.06	1.00	0.87	1.26	1.71	1.37
Housing Services	2.08*	1.80*	2.07*	1.93*	1.86*	**2.15**
Sobering Services	1.97	2.69	1.62	1.27	1.79	1.31
Asthma remediation services	1.26	2.70	2.17	1.21	1.70	2.08
Manager (vs. senior leader)	**2.45**	**2.22**	**2.85**	**1.93**	**1.76**	**2.01**
Frontline (vs. senior leader)	**2.74**	**2.89**	**3.14**	**3.35**	1.93	1.92

Bold indicates OR significant at *p* < 0.05

*Indicates this result was not robust to sensitivity analysis with mixed effects at the level of county (p not significant in sensitivity analysis)

In all six multivariate models, CalAIM participation was strongly associated with increased odds of perceived improvement (OR 2.28 to 4.97), as was being a housing services provider (OR 1.80 to 2.15). Being a manager and frontline staff was associated with increased odds of perceived improvement compared to senior leaders in all/most models (Manager OR 1.76 to 2.85; frontline OR 1.92–3.35). In the sensitivity analysis with mixed effects clustered on county, being a housing provider and a non-profit organization were no longer significant in some outcomes (as indicated in Table 2) while all other variables maintained *p* < 0.05.

## Discussion

We conducted a statewide survey of SSOs familiar with CalAIM eighteen months into implementation of California’s major Medicaid HRSN initiative, CalAIM. We achieved a sample of 355 respondents, from 88% of counties, a mix of larger and smaller organizations, in frontline through senior leadership roles. The results shed light on our two research questions.

### Smaller SSOs may be less likely to participate in calaim, mediated by disproportionately low pilot participation

We found some indications that among SSOs familiar with CalAIM, smaller organizations may choose to participate at lower rates than larger SSOs. Organizations that operate in only one county were less likely to participate in CalAIM, and organizations with fewer than 50 FTEs and that are non-profits (vs. private or government entities) were less likely to participate in the pilot programs, which in turn was strongly associated with CalAIM participation. Our results are congruent with the Wong et al. analysis of tax forms of CalAIM contracted SSOs, which found higher than expected median revenue of contracted SSOs compared to average SSO revenues [5]. Our findings suggesting that there were disparities in pilot participation which in turn affected CalAIM participation, highlight the potential inequity created when a full-scale implementation builds from pilots, as pilots may include a biased subset of easier-to-engage and more highly resourced organizations.

Our findings that pilot participation was strongly associated with CalAIM participation, and that having had prior MCP contracts and prior experience with an HIE or CIE was moderately associated with pilot participation suggest that prior experience played a substantial role in CalAIM participation decisions at this stage of implementation. In addition to dedicated catch-up efforts to support non-pilot participating SSOs, explicitly striving for inclusion of a diverse set of SSOs from the start might mitigate this effect.

We did not find evidence that organizations that specialize in serving BIPOC/NEL populations are less likely to participate or that they have different perceptions about CalAIM’s impacts. This was in contrast to our hypothesis based on expert opinion and focus groups and may be because our survey question did not adequately assess BIPOC/NEL specialization. Of respondents reporting any BIPOC specialization, 70% (*n* = 73 of 104) reported that they specialized in more than one BIPOC group, indicating that respondents may have interpreted the question to mean “do you serve these populations”. However, even when we conducted sensitivity analysis defining BIPOC/NEL specialization as only respondents who specialized in only one BIPOC group, we did not find any differences in CalAIM participation or in perceptions of impacts (although this may have been too small a subset [*n* = 31] to detect an association). It is also possible that our null results on this question reflect selection bias in the survey respondents, such that organizations specializing in BIPOC/NEL populations and not participating in CalAIM were less likely to respond to the survey, and/or more likely to be excluded by the criterion of lack of familiarity with CalAIM. More research is needed to ascertain whether BIPOC/NEL-serving organizations are participating equally in CalAIM.

### SSOs participating in CalAIM perceive improvement in health care-social services integration, especially among frontline staff

SSO respondents perceived improvements across all six dimensions of health care-social services integration tested (at the individual patient level, organizational level, and in terms of SSO IT infrastructure and finances). Perceived improvement was strongly associated with CalAIM participation, indicating that contracted organizations are indeed seeing benefit from their participation. Interestingly, frontline staff and managers were more likely to report improvements than senior leaders. This may be because frontline and managerial staff are closer to on-the-ground improvements in the availability of services and resources to offer patients with high needs, while senior leaders bear the burden of administrative and financial frustrations. Housing providers also had higher odds of perceived improvement compared to providers of other service types, likely because housing supports have been a major focus of the state and MCPs in initial CalAIM implementation—this result was not robust to sensitivity analysis that accounted for county-level mixed effects, suggesting that some of the housing provider effect may have been concentrated in certain counties and/or within multiple respondents from the same SSO.

There was no consistent indication that smaller SSOs perceived improvements differently than larger SSOs. While SSOs with fewer than 50 FTEs had higher odds of perceived improvement in individual patient-level coordination on two dimensions, non-profit status was associated with lower odds of perceived improvement in one of these same dimensions (patient access to coordinated services), and in lower odds of improved IT infrastructure. No other indicator of small SSOs had a significant association with perceived improvement in any direction, nor did specializing in BIPOC/NEL populations.

### Feasibility of broad SSO surveys

Our survey experience revealed a feasible method for states to monitor and elevate SSO perspectives during rollout of HRSN initiatives. We reached a large sample of both participants and non-participants that included a mix of counties, SSO sizes, and service types. Descriptive results were rapidly shared with state officials, followed by the analysis presented herein.

### Limitations

This analysis was intended to understand whether SSO participation in CalAIM was potentially inequitable and elevate SSO implementation perspectives. While we achieved the goal sample size of at least 350 organizations and recruited a broad sample reflecting a mix of SSOs across many dimensions, without a comprehensive list of SSOs in the state (which is unavailable at this time despite ongoing conversations in the research and implementation community), we were unable to ascertain the survey’s representativeness. Since organizations with “no familiarity” with CalAIM were excluded from the survey, the results reflect those organizations familiar with CalAIM and whether or not they chose to participate and would not generalize to SSOs who were not aware of CalAIM.

The survey was also likely to include organizations that are more connected and therefore who would have received reminders and survey links on multiple listservs and other technical assistance venues. Despite the likely overrepresentation of these savvier organizations, we had sufficient sample size to find significant associations suggesting that smaller SSOs had higher and unique barriers to CalAIM participation. We had the possibility of multiple respondents from a single SSO, which would lead to non-random errors and artificially low *p*-values; reassuringly the main results stood in our sensitivity analysis at the level of county, however we were unable to cluster at the level of SSO due to the data collected. As described, the survey question for BIPOC/NEL may have been misinterpreted by participants, limiting our ability to gauge specific experiences of BIPOC/NEL-specializing SSOs. Finally, while a useful tool for rapid collecting a broad array of SSO experiences, all surveys are limited to respondents’ self-report and opinions. This instrument was not validated (no validated alternatives exist); we piloted with 4 SSO employees but this would not be sufficient to assess validity and reliability. Surveys restrict respondents to pre-determined questions and answers. The results should be interpreted as suggestive and hypothesis generating.

### Future research

Next steps to round out the full picture of SSO experiences under CalAIM include further qualitative study (i.e. a follow-up survey; dedicated interviews with smaller and BIPOC-specializing SSOs), and quantitative analysis (i.e. of claims and EHR patient outcome data). Our experience points to a priority need to generate a comprehensive landscape dataset of SSOs, in order to ascertain representativeness of SSOs in both research and implementation efforts.

## Conclusions

This survey provided a window into the SSO experience, eighteen months into implementation of a major HRSN Medicaid initiative. We found some indications that among SSOs familiar with CalAIM, smaller organizations were less likely to choose to participate, possibly mediated by lower rates of prior pilot participation. People working in SSOs, especially those who were contracted CalAIM participants and frontline/managerial respondents, reported improvements in health care-social services integration across several dimensions. Our results did not reveal a distinct experience of BIPOC/NEL specializing SSOs, positively or negatively. These results can help inform implementation of similar HRSN efforts in other states.

## Electronic supplementary material

Below is the link to the electronic supplementary material.

Supplementary Material 1

## Data Availability

The datasets generated during and/or analysed during the current study are not publicly available due proprietary ownership by the California Health Care Foundation but are available from the corresponding author on reasonable request. Detailed descriptive information of the full survey (including but not limited to the SSO respondent subset) are publicly available in a California Health Care Foundation report (URL below). The present work is new in that it provides previously unpublished detailed description of the SSO subset, and analysis using regression methods to answer the specific research questions, neither of which were presented in the publicly available report. https://www.chcf.org/publication/calaim-experiences-implementer-views-18-months-reforms/#related-links-and-downloads.

## Abbreviations

BIPOC: Black Indigenous People of Color
CalAIM: California Advancing and Innovating Medi-Cal
ECM: Enhanced Care Management
EHR: Electronic Health Record
FTE: Full time employee
HIE/CIE: Health Information Exchange, Community Information Exchange
HRSN: Health Related Social Needs
MCP: Managed Care Plan
NEL: Non-English Language
SSO: Social Services Organization
