# Supplementary material for: Social service organizations report improvements in social services-health care integration in survey during California’s medicaid initiative (“CalAIM”)
Source: BMC Public Health. 2025 Jul 2;25:2234. doi: 10.1186/s12889-025-23419-3 (PMC12220213; doi:10.1186/s12889-025-23419-3)
Supplement: Supplementary file 1 — Supplementary Material 1 [file 12889_2025_23419_MOESM1_ESM.docx]

Appendix Table 1. Full Text of relevant SSO survey questions used in analysis

| **Organizational and respondent characteristics** | |
| --- | --- |
| Single county | Which of the following California counties do you operate in? **You may select all that apply.** If you do not operate in California, please indicate that. *[MULTI-CHOICE CHECKLIST OF CA COUNTIES WITH EXCLUSIVE ANSWER CHOICES FOR: “Statewide” “Do not operate in California”]* |
| FTEs | Approximately how many FTEs (Full Time Equivalents) does your organization have? Please count the FTEs for your entire organization. *[<10, 10-24, 25-49, 50-99, 100-249, 250+, Unsure]* |
| Nonprofit status | Is your employer a private entity, a non-profit, a government agency, or something else? [*Please select all that apply. Private entity, non profit, government agency]* |
|  |  |
| BIPOC/NEL specialization  (in final analysis, a binary variable for BIPOC/NEL specialization included if the respondent selected any of Asian American, Pacific Islander, Black, Latino/x, Native American, and Populations whose primary language isn’t English). | Does your organization specialize in serving any of the following populations? By specialize, we mean that you provide services specifically tailored to those populations’ unique needs. Please select all that apply. If you do not specialize, please select “We don’t specialize in a specific population.” *[Children and youth, Older adults, Asian American populations, Pacific Islander populations, Black populations, Latino/x populations, Native American populations, Populations whose primary language isn’t English, Other, We don’t specialize in a specific population, Unsure]* |
| Service type | What kind of services does your organization provide? Please select all that apply. *[Housing or homeless services, Recuperative care/Medical respite, Food-related services/Food assistance, Sobering center/Sobering services, Services for older adults or people with disabilities to live in the community, General social services assistance, Benefits navigation, Re-entry services following incarceration, Home modification services, Asthma remediation services, Child welfare services, Legal services, Information & Referral services]* |
| Respondent role  (in final analysis “Program level management” and “Manager of frontline staff” were combined into a single “Manager” category) | Which of the following best describes your job title or role? [*Senior leadership (e.g., CEO, COO, CMO, Executive Director), Program level management (e.g., program director or manager), Manager or supervisor of frontline staff, Frontline staff or provider (e.g., clinician, case manager), Other, Unsure]* |
| Pilot Participation | Did your organization previously participate in Whole Person Care or Health Homes? Please select all that apply. *[Yes, participated in Whole Person Care, Yes, participated in Health Homes,*  *No, did not participate in either Whole Person Care or Health Homes, Unsure]* |
| Prior MCP Contracting  Prior HIE/CIE Experience  (Binary variable combining A lot, A fair amount, or A little considered as a positive response) | Prior to CalAIM’s launch (January 2022), how much experience did your organization have with the following: [*A lot, A fair amount, A little, None, Unsure]*  Contracting with at least one Medi-Cal managed care plan (MCP)  Contributing client data to a centralized data repository or warehouse, (e.g., health information exchange (HIE) or community information exchange (CIE)) |
| **Outcomes** | |
| CalAIM Participation | Is your organization currently providing Enhanced Care Management (ECM) or Community Supports (CS)? Please select all that apply. If your organization is NOT currently providing ECM or Community Supports (even if you did provide ECM/CS previously or are planning to in the future), please indicate that. [*ECM, Community Supports, Neither, Unsure]* |
| Perceptions of improvement in :   1. Patient access to services (inc. social needs) 2. Patient coordination of services | Thinking about the experiences of the people you serve (e.g., patients, members, or clients), please indicate whether you personally think the experiences of the following have gotten better or worse as a result of CalAIM’s implementation—or if they have stayed about the same. If you are unsure, just select that.  *[Much better, Somewhat better, Stayed about the same, Somewhat worse, Much worse, Not applicable, Unsure]*   1. Overall access to services, including those that address health-related social needs (e.g., housing navigation, medically supported food and nutrition services) 2. Coordination of services, including those that address health-related social needs |
| Perceptions of improvement in:   1. SSO’s ability to manage patients’ needs 2. SSO’s coordination with other organizations 3. SSO’s IT infrastructure 4. SSO’s financial stability | Now thinking about your own organization, please indicate whether you personally think each of the following has gotten better or worse as a result of CalAIM—or if it has stayed about the same.  There are a lot of different parts to CalAIM, but try to think about the program as a whole and how things have changed since implementation began.  *[Much better, Somewhat better, Stayed about the same, Somewhat worse, Much worse, Not applicable, Unsure]*   1. Your organization’s ability to manage the comprehensive needs of the people you serve 2. Your organization’s ability to coordinate with other organizations serving the same people 3. Your organization’s IT/software capacity and infrastructure 4. Your organization’s financial stability |

Appendix Table 2. Multivariate analysis of factors associated with higher odds of Pilot Participation (Whole Person Care and/or Health Homes) among organizations operating in a single county, in counties that were pilot counties (n=209).

| Organizational Characteristic | OR (95% CI) |
| --- | --- |
| Under 50 FTEs (vs. >=50) | **0.42 (0.19-0.94)** |
| Nonprofit org (vs private/govt.) | **0.15 (0.04-0.54)** |
| Any BIPOC/NEL specialty (vs. none) | 0.94 (0.43-2.02) |
| Housing services | **12.4 (3.53-43.61)** |
| Services of community living | **3.11 (1.47-6.55)** |
| Sobering services | 1.25 (0.32-4.95) |
| Asthma remediation services | 1.17 (0.05-23.4) |

Bold indicates statistically significant at p<0.05.

Appendix Table 3. Significant associations in bivariate analysis chi-square testing of independent variables with six outcomes relating to perceptions of improved health care-social services integration (p-values).

|  | Patient access to services (inc. social needs) | Patient coordination of services | SSO’s ability to manage patients’ needs | SSO’s coordination with other organizations | SSO’s IT infrastructure | SSO’s financial stability |
| --- | --- | --- | --- | --- | --- | --- |
| Small/Community-Ties Indicators | | | | | | |
| *Under 50 FTEs* |  |  |  |  |  |  |
| *SSO operates in 1 county only* | <0.05 | <0.05 |  |  | <0.05 | <0.05 |
| *Nonprofit* | <0.05 | <0.05 | <0.05 | <0.05 | <0.05 | <0.05 |
| *Any BIPOC/NEL specialty* |  |  |  |  |  |  |
| SSO experience |  |  |  |  |  |  |
| *Pilot participant* | <0.05 | <0.05 | <0.05 | <0.05 | <0.05 | <0.05 |
| *Prior HIE/CIE participation* | <0.05 | <0.05 | <0.05 | <0.05 | <0.05 | <0.05 |
| *CalAIM Participant* | <0.05 | <0.05 | <0.05 | <0.05 | <0.05 | <0.05 |
| Services provided |  |  |  |  |  |  |
| *Housing/Homelessness* | <0.05 | <0.05 | <0.05 | <0.05 | <0.05 | <0.05 |
| *Food-related services/Food assistance* |  |  | 0.08 | <0.05 |  |  |
| *Information & Referral services* |  |  |  |  | 0.06 |  |
| *General social services assistance* |  |  |  |  |  |  |
| *Benefits navigation* |  |  | <0.05 | <0.05 |  |  |
| *Services for older adults or people with disabilities to live in the community* | 0.07 |  |  |  | <0.05 | <0.05 |
| *Recuperative care/Medical respite* | <0.05 | <0.05 | <0.05 | <0.05 | <0.05 | <0.05 |
| *Re-entry services following incarceration* |  | 0.09 | <0.05 |  |  | 0.08 |
| *Child welfare services* |  |  |  | 0.09 | 0.06 | <0.05 |
| *Home modification services* |  |  |  |  |  |  |
| *Sobering center/Sobering services* | <0.05 | <0.05 | <0.05 | <0.05 | <0.05 | <0.05 |
| *Legal services* |  |  |  |  |  |  |
| *Asthma remediation services* | <0.05 | <0.05 | <0.05 | <0.05 | <0.05 | <0.05 |
|  |  |  |  |  |  |  |
| Respondent role type | <0.05 | <0.05 | <0.05 | <0.05 | <0.05 | <0.05 |

Blank indicates p-value>0.1
